# Ambient nitrogen dioxide in 47 187 neighbourhoods across 326 cities in eight Latin American countries: population exposures and associations with urban features

**DOI:** 10.1016/S2542-5196(23)00237-1

**Published:** 2023-12-04

**Authors:** Josiah L Kephart, Nelson Gouveia, Daniel A Rodríguez, Katherine Indvik, Tania Alfaro, José Luis Texcalac-Sangrador, J Jaime Miranda, Usama Bilal, Ana V Diez Roux

**Affiliations:** a Urban Health Collaborative, Dornsife School of Public Health, Drexel University, Philadelphia, PA, USA; b Department of Environmental and Occupational Health, Dornsife School of Public Health, Drexel University, Philadelphia, PA, USA; c Department of Epidemiology and Biostatistics, Dornsife School of Public Health, Drexel University, Philadelphia, PA, USA; d Department of Preventive Medicine, University of São Paulo Medical School, São Paulo, Brazil; e Department of City and Regional Planning and Institute for Transportation Studies, University of California, Berkeley, CA, USA; f Escuela de Salud Pública, Facultad de Medicina, Universidad de Chile, Santiago, Chile; g Department of Environmental Health, Center for Population Health Research, National Institute of Public Health, Cuernavaca, Mexico; h CRONICAS Centre of Excellence in Chronic Diseases, Universidad Peruana Cayetano Heredia, Lima, Peru; i The George Institute for Global Health, University of New South Wales, Sydney, NSW, Australia

## Abstract

**Background:**

Health research on ambient nitrogen dioxide (NO_2_) is sparse in Latin America, despite the high prevalence of NO_2_-associated respiratory diseases in the region. This study describes within-city distributions of ambient NO_2_ concentrations at high spatial resolution and urban characteristics associated with neighbourhood ambient NO_2_ in 326 Latin American cities.

**Methods:**

We aggregated estimates of annual surface NO_2_ at 1 km^2^ spatial resolution for 2019, population counts, and urban characteristics compiled by the SALURBAL project to the neighbourhood level (ie, census tracts). We described the percentage of the urban population living with ambient NO_2_ concentrations exceeding WHO air quality guidelines. We used multilevel models to describe associations of neighbourhood ambient NO_2_ concentrations with population and urban characteristics at the neighbourhood and city levels.

**Findings:**

We examined 47 187 neighbourhoods in 326 cities from eight Latin American countries. Of the roughly 236 million urban residents observed, 85% lived in neighbourhoods with ambient annual NO_2_ above WHO guidelines. In adjusted models, higher neighbourhood-level educational attainment, closer proximity to the city centre, and lower neighbourhood-level greenness were associated with higher ambient NO_2_. At the city level, higher vehicle congestion, population size, and population density were associated with higher ambient NO_2_.

**Interpretation:**

Almost nine out of every ten residents of Latin American cities live with ambient NO_2_ concentrations above WHO guidelines. Increasing neighbourhood greenness and reducing reliance on fossil fuel-powered vehicles warrant further attention as potential actionable urban environmental interventions to reduce population exposure to ambient NO_2_.

**Funding:**

Wellcome Trust, National Institutes of Health, Cotswold Foundation.

## Introduction

Ambient nitrogen dioxide (NO_2_) is a ubiquitous urban air pollutant produced by fossil fuel combustion. NO_2_ is emitted by outdoor and indoor point sources, such as industrial processes and household cooking and heating,^1^ and through mobile sources, such as exhaust from fossil fuel-powered vehicles.^1^ Under certain atmospheric conditions, NO_2_ can rapidly transform to other chemical structures and is a key ingredient in the formation of ground-level ozone.^1^ Because of NO_2_'s tendency to rapidly transform over time and space, ambient NO_2_ concentrations can have highly granular spatial variability within cities.^2^, ^3^ This variability is often linked to spatially varying social characteristics, resulting in within-city social disparities in NO_2_ exposures.^4^

Historically, epidemiological research on NO_2_ has faced the challenge of examining NO_2_ exposure within complex mixtures of co-occurring pollutants, such as exhaust from fossil fuel-powered vehicles, and many epidemiological analyses have approached ambient NO_2_ as a proxy for traffic-related air pollutant mixtures.^3^ However rapidly growing evidence supports increased attention to the role of NO_2_ itself as an independent risk factor for health.^5^ Exposure to NO_2_ contributes to respiratory disease^1^ and all-cause mortality,^6^, ^7^ among other health effects.^1^ Children, older adults, and individuals with respiratory disease are particularly susceptible to the health effects of NO_2_ exposure.^1^ In 2021, WHO lowered the air quality guideline for annual NO_2_ by 75% (from 40 μg/m^3^ to 10 μg/m^3^), citing growing evidence of the impacts of NO_2_ on health.^8^ The updated WHO air quality guidelines and, specifically, the substantial reductions in guidelines for NO_2_ warrants renewed attention towards who is exposed to harmful ambient NO_2_ concentrations and how ambient NO_2_ can be reduced in urban settings that remain highly dependent on fossil fuels for transit and industry.

To date, limited research has examined population exposures to ambient NO_2_ and urban factors associated with ambient NO_2_ in Latin America. Even fewer studies have examined within-city differences in NO_2_ concentrations in the region. However, Latin America has both high urbanisation (80% of the population lives in urban areas)^9^ and a high prevalence of NO_2_-associated respiratory diseases.^10^ A 2019 study of asthma incidence attributable to ambient NO_2_ found that Lima, Peru, and Bogotá, Colombia were among the top three cities globally for asthma incidence attributable to NO_2_ exposure.^10^ A 2022 study of 968 urban areas in Latin America estimated that 16% of paediatric asthma cases in Latin American cities are attributable to ambient NO_2_ exposures.^11^ Despite the substantial health impacts of ambient NO_2_ in the region, ambient NO_2_ monitoring networks in the region are sparse.^12^ However, recent advances in satellite-derived global estimates of surface NO_2_ at fine spatial resolution^11^ provide novel opportunities to examine social disparities and spatial variations in population exposures to NO_2_ within and between cities in this highly urbanised region.

**Research in context**

**Evidence before this study**

We searched PubMed and Google Scholar for articles in all languages published from the inception of the databases until April 2, 2023. We used the following keywords: (“nitrogen dioxide” OR “NO_2_”) AND (“built environment” OR “Latin America” OR “population exposure” OR “urban form” OR “neighbourhood” OR “disparities”). Recent global studies conducted at the city level identify Latin America as a region with a high prevalence of paediatric asthma attributable to ambient nitrogen dioxide (NO_2_). NO_2_ concentrations are particularly localised in nature, and previous studies have reported within-city disparities in ambient NO_2_ concentrations in the USA and Europe. However, there is limited knowledge about within-city differences in NO_2_ concentrations, population exposure, and associated neighbourhood features within Latin America or the Global South more broadly, where urban features are distinct from better-studied high-income settings.

**Added value of this study**

In this study, we investigated within-city differences in ambient NO_2_ among 47 187 neighbourhoods across 326 cities in eight Latin American countries. The study included approximately 236 million urban residents observed, of whom 85% lived in neighbourhoods with ambient NO_2_ above WHO annual guidelines. We found that higher neighbourhood-level educational attainment, closer proximity to the city centre, and lower neighbourhood-level greenness were associated with higher ambient NO_2_. At the city level, higher levels of vehicle congestion, population size, and population density were associated with higher ambient NO_2_. This study is the first to describe disparities in ambient NO_2_ exposure within cities and to examine the relationship between urban features and NO_2_ levels across a large number of cities in a region in the Global South.

**Implications of all the available evidence**

The existing literature suggests that Latin American cities have a high burden of ambient NO_2_-related health effects and that within-city disparities in ambient NO_2_ are common in high-income countries. Our study offers detailed evidence on population exposure to NO_2_ at the neighbourhood level in a large number of Latin America cities, highlighting distinct patterns of NO_2_ population exposure that differ from those found in high-income countries. Additionally, we identified neighbourhood and city features that could potential be targeted as interventions to reduce NO_2_ exposure in Latin American cities.

To address these knowledge gaps on population exposures to ambient NO_2_ in Latin America and the relationship between NO_2_ and the urban environment, this study aims to describe population exposures to ambient NO_2_ and urban characteristics associated with differences in ambient NO_2_ exposure at the census tract level within 326 Latin American cities.

## Methods

### Study setting

This study was conducted as part of the *Salud Urbana en América Latina* (SALURBAL) project. The SALURBAL study protocol was approved by the Drexel University Institutional Review Board (ID number 1612005035). This international scientific collaboration has compiled and harmonised data on social, environmental, and health characteristics for hundreds of cities in 11 Latin American countries.^13^ Cities in SALURBAL are composed of clusters of administrative units (ie, municipalities) encompassing the visually apparent urban built-up area as identified using satellite imagery.^14^ Cities were defined as all urban agglomerations within the 11 countries that contained more than 100 000 residents as of 2010,^14^ facilitating examination of a diverse set of cities, from small cities to megacities. The SALURBAL project previously published an analysis of the variability and predictors of ambient fine particulate matter (PM_2·5_) across the Latin American region,^15^ focusing on larger administrative units (ie, municipalities).

In this analysis, we examine neighbourhood-level ambient NO_2_ in 326 cities in Argentina, Brazil, Chile, Colombia, Costa Rica, Guatemala, Mexico, and Panama (appendix pp 3–10). Cities in El Salvador, Nicaragua, and Peru were excluded due to the unavailability of data at the neighbourhood level. Neighbourhood administrative units varied in name and official definition by country. We used the country-specific, small-area administrative units most analogous to US census tracts, henceforth referred to as “neighbourhoods”. Detailed information on the administrative units and census used for each country is available in the appendix (p 2). Across countries, these neighbourhoods had a median population of 2063 and a median area of 0·34 km^2^ (equivalent to a square with 0·58 km sides).

### Neighbourhood NO_2_ exposures

We used estimates published in 2022 of annual surface NO_2_ at 1 km^2^ spatial resolution.^11^ These estimates were based on a previous land use regression model of mean surface NO_2_ from 2010 to 2012 at 100 m resolution by Larkin and colleagues.^16^ Along with satellite products and land use information, Larkin and colleagues incorporated 105 air monitors from our study countries of Brazil, Chile, and Colombia out of a total of 5220 monitors globally. Despite the smaller representation of air monitors, this model performed better in South America (adjusted *R*^2^=0·63) than in North America (adjusted *R*^2^=0·52), Europe (adjusted *R*^2^=0·52), or any other world region. The land use regression estimates were subsequently adjusted for bias in rural areas using chemical transport models and scaled to an extended timeframe (annual means from 2005 to 2020) using satellite NO_2_ columns from the Ozone Monitoring Instrument version 4.0 product.^11^ For this analysis, we used NO_2_ estimates from the year 2019, the most recent year before the pronounced changes in ambient air pollution associated with the COVID-19 pandemic.^17^ To estimate neighbourhood annual mean NO_2_, we averaged the values of all NO_2_ raster grid cells overlapping or contained within the neighbourhood spatial boundary, area-weighting for the proportion of each grid cell contained within the neighbourhood boundary. The resulting output was an estimate of annual surface NO_2_ in the year 2019 for each neighbourhood in the study area.

### Neighbourhood and city characteristics

We used data on neighbourhood characteristics and population compiled from national census bureaus and other sources by the SALURBAL project.^14^ We used the most recent available census for each country; information on the year of each census used is available in the appendix (p 2). At both the neighbourhood and city levels, the SALURBAL project previously estimated population density (population divided by built-up area), educational attainment (percentage of the population aged 25 years or older who completed primary education or above), intersection density (density of the set of nodes with more than one street emanating from them per km^2^ of built-up area), and area median greenness measured by the normalised difference vegetation index (NDVI). At the neighbourhood level, we also calculated distance from the city centre as the Euclidean distance (km) between the neighbourhood centroid and city hall. At the city level, we also estimated city population, gross domestic product (GDP) per capita (computed as purchasing power parities in constant 2011 international US dollars of each city in 2015 using estimates from the first subnational administrative level, typically equivalent to departments or states^18^), and city-level traffic congestion (increase in road vehicle travel time due to congestion in the street network^19^). Detailed information on urban features is presented in the appendix (p 14).

### Statistical analysis

We calculated summary statistics and boxplots of neighbourhood NO_2_ concentrations, overall and stratified by country. Due to the small number of cities represented by each country, we pooled cities from Costa Rica (n=1 city), Guatemala (n=2), and Panama (n=3) into a single country grouping for Central America for all analyses. For comparability with the NO_2_ data source, we transformed the WHO annual guideline from μg/m^3^ to parts per billion (ppb; 10 μg/m^3^ is approximately 5·3 ppb) under standard assumptions (atmospheric pressure at sea level and 25°C temperature). We created summary statistics of the population living in neighbourhoods with ambient NO_2_ concentrations above and below annual WHO air quality guidelines (10 μg/m^3^), overall and by country. We summarised city-level mean NO_2_ for descriptive analysis by aggregating neighbourhood NO_2_ estimates to the city-level using a population-weighted average.

To estimate between-country versus between-city versus within-city variation in neighbourhood ambient NO_2_ exposures, we used a mixed effects one-way ANOVA with random intercepts for city and country.

We used multilevel univariable and multivariable models to describe associations between neighbourhood-level ambient NO_2_ concentrations and population and urban characteristics at the neighbourhood and city levels. All independent variables were operationalised as Z scores of the overall distribution of 47 187 neighbourhoods for each respective variable. We first conducted a univariable analysis of each independent variable and the dependent variable of neighbourhood annual NO_2_. We then assessed all variables for collinearity using Spearman correlation coefficients. Finally, we modelled all neighbourhood-level and city-level predictors together as a single multilevel model. The percent change in variance between empty and multivariable models was calculated to describe the total variance explained by the multivariable model. All univariable and multivariable models were adjusted for country as a fixed effect and city as a random intercept. As a sensitivity analysis, we used the same multivariable model but replaced NO_2_ and urban feature data in or closest to 2019 with NO_2_ and urban feature data in or closest to each country's census year. To evaluate between-country differences in these associations, we also stratified the main model by country for Brazil, Argentina, Chile, Colombia, and Mexico. We did not include the Central American countries of Guatemala, Costa Rica, and Panama in this sensitivity analysis due to the small number of cities (≤3) in these countries.

Data processing and analyses were conducted in R version 4.1.0.

### Role of the funding source

The funders of the study had no role in study design, data collection, data analysis, data interpretation, or writing of the report.

## Results

**Figure 1:**
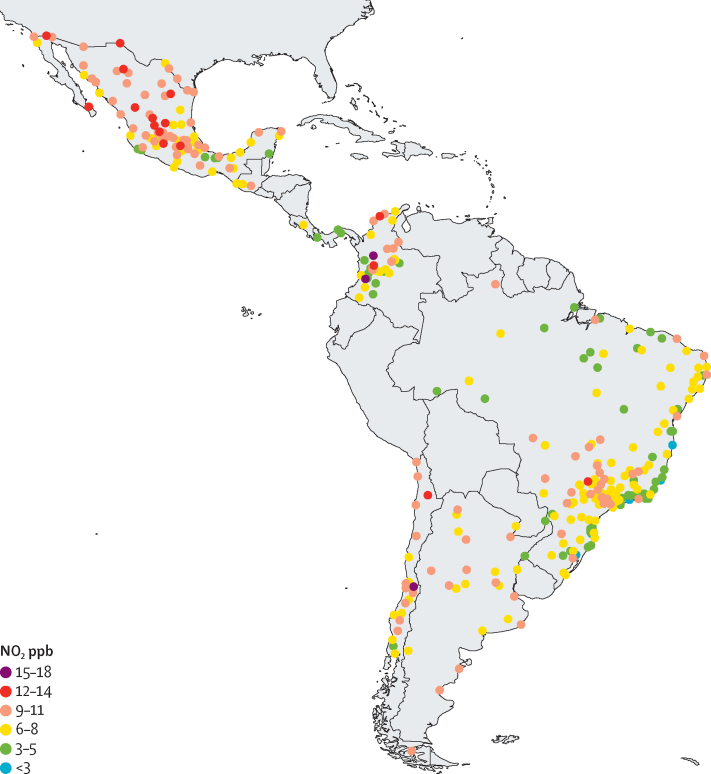
Location of study cities (n=326) and city-level population-weighted annual concentration of ambient NO_2_ in 2019 The WHO guideline for annual NO_2_ is 10 μg/m^3^ (approximately 5·3 ppb) and all neighbourhoods that exceed this guideline are represented by yellow, orange, red, or purple. NO_2_=nitrogen dioxide. ppb=parts per billion.

We examined 47 187 neighbourhoods in 326 cities in eight Latin American countries (table 1, appendix pp 3–10). The geographical locations of observed cities are presented in figure 1. Within study cities, urban neighbourhoods in the pooled Central American group (Guatemala, Costa Rica, and Panama; median NDVI 0·59 [IQR 0·43–0·75]) and Brazil (0·57 [0·41–0·75]) were greenest, while neighbourhoods in Chilean cities were the least green (0·32 [0·20–0·46]). Neighbourhoods in Colombia had the highest population density (median 12·7 thousand residents per km^2^ [IQR 4·4–21·3]) while neighbourhoods in Brazil were the least dense (3·9 [1·3–8·4]). At the city level, cities in the Central American group had the greatest traffic congestion (median 36% [IQR 20–60] longer trip duration than free-flow conditions due to congestion) while cities in Brazil were the least congested (9% [6–11] longer trip duration due to congestion). Neighbourhood and city population and characteristics are described overall and by country in table 1.

**Table 1:** Population characteristics and urban form of study neighbourhoods (n=47 187) within 326 Latin American cities

	**Total**	**Argentina**	**Brazil**	Central American*	**Chile**	**Colombia**	**Mexico**
**Neighbourhood level**							
Number of neighbourhoods	47 187	2044	4004	5563	744	3064	31 768
Population density†	5·7 (2·2–11·2)	6·5 (2·5–9·9)	3·9 (1·3–8·4)	6·1 (2·1–14·5)	8·0 (4·5–12·4)	12·7 (4·4–21·3)	5·5 (2·2–10·4)
Education‡	89·7 (81·8–94·8)	90·8 (84·7–95·7)	67·4 (60·2–75·1)	78·6 (58·6–90·5)	91·6 (86·9–95·0)	81·5 (68·9–89·5)	91·7 (87·1–95·9)
Intersection density§	132 (72–199)	87 (59–108)	88 (38–129)	76 (19–149)	170 (109–242)	111 (23–333)	154 (96–213)
Greenness (NDVI)	0·45 (0·31–0·62)	0·41 (0·30–0·60)	0·57 (0·41–0·75)	0·59 (0·43–0·75)	0·32 (0·20–0·46)	0·51 (0·39–0·70)	0·41 (0·28–0·57)
Distance from city centre (km)	8·4 (4·2–15·8)	15·7 (5·8–29·5)	10·3 (4·4–19·9)	9·3 (5·8–14·7)	4·8 (2·7–10·3)	4·7 (2·3–9·0)	8·2 (4·3–15·7)
**City level**							
Number of cities	326	22	152	6	21	33	92
City population (thousands)	307 (181–643)	332 (194–628)	255 (165–567)	1153 (253–2424)	243 (174–382)	369 (165–628)	396 (232–887)
GDP per capita (US$ thousands)	14·8 (10·5–20·6)	19·6 (11·3–22·3)	19·3 (8·6–21·0)	15·8 (11·8–25·1)	17·7 (13·0–26·6)	11·8 (8·9–13·8)	14·0 (11·5–17·6)
Traffic congestion index¶	11% (8–19)	10% (9–13)	9% (6–11)	36% (20–60)	27% (20–32)	32% (27–39)	13% (10–20)
Population density†	6·8 (5·5–8·9)	5·5 (5·1–6·8)	6·2 (5·3–8·0)	7·9 (7·5–8·4)	7·1 (6·6–9·0)	15·9 (13·1–18·9)	6·5 (5·6–7·8)
Education‡	73·0 (65·7–82·2)	79·8 (77·9–81·8)	65·7 (61·6–69·7)	86·7 (79·0–90·1)	89·8 (87·8–91·6)	83·7 (81·7–85·9)	79·6 (74·4–83·0)
Intersection density§	87·9 (74·3–107·8)	81·2 (72·8–95·1)	82·2 (72·6–93·7)	60·9 (55·5–67·8)	120·5 (114·7–133·8)	121·6 (104·5–135·7)	92·9 (76·8–107·6)
Greenness (NDVI)	0·82 (0·76–0·86)	0·79 (0·70–0·85)	0·82 (0·79–0·87)	0·88 (0·85–0·90)	0·75 (0·29–0·81)	0·86 (0·84–0·87)	0·76 (0·61–0·85)

Data are n or median (IQR). NDVI=normalised difference vegetation index. GDP=gross domestic product.

* Central American grouping includes urban neighbourhoods in Costa Rica (n=1 city), Guatemala (n=2), and Panama (n=3).

† Thousands of residents per square kilometre.

‡ Percentage of the population aged 25 years or older who completed primary education or above.

§ Intersections per square kilometre of built-up area.

¶ Percent longer trip duration due to traffic congestion, as a percentage of trip time without congestion.

**Figure 2:**
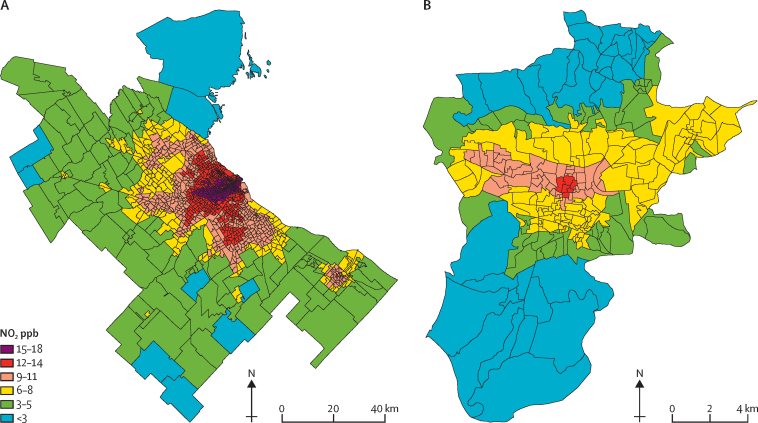
Within-city variation in neighbourhood-level ambient NO_2_ in two selected cities with varying population and geographical sizes (note the panel-specific scale bars) Black lines represent neighbourhood boundaries and colours represent annual mean ambient NO_2_ in 2019. (A) The metropolitan area of Buenos Aires, Argentina (population approximately 16 million). (B) Quetzaltenango, Guatemala (population approximately 295 000). In both cities, neighbourhood NO_2_ concentrations trend higher with greater proximity to the urban core. The WHO guideline for annual NO_2_ is 10 μg/m^3^ (approximately 5·3 ppb) and all neighbourhoods that exceed this guideline are represented by yellow, orange, red, or purple. NO_2_=nitrogen dioxide. ppb=parts per billion.

In figure 1, we present the population-weighted city-level mean NO_2_ for context (as distinct from the primary analysis of neighbourhood-level concentrations). City-level NO_2_ concentrations appear to have strong variation between many cities in close proximity, suggesting the importance of local drivers of ambient NO_2_. In figure 2, we present neighbourhood-level NO_2_ for two selected cities of varying sizes: the metropolitan area of Buenos Aires, Argentina (population of around 16 million; figure 2A) and Quetzaltenango, Guatemala (population of around 295 000; figure 2B). In both selected cities, as typical across study cities, neighbourhood NO_2_ concentrations trend higher with greater proximity to the urban core. Consistent with the overall findings of Figure 1, Figure 2, our variance decomposition model showed that 9·4% of total variance in neighbourhood NO_2_ was between countries, 30·3% of variance was between cities, and 60·3% of variance was within cities.

**Figure 3:**
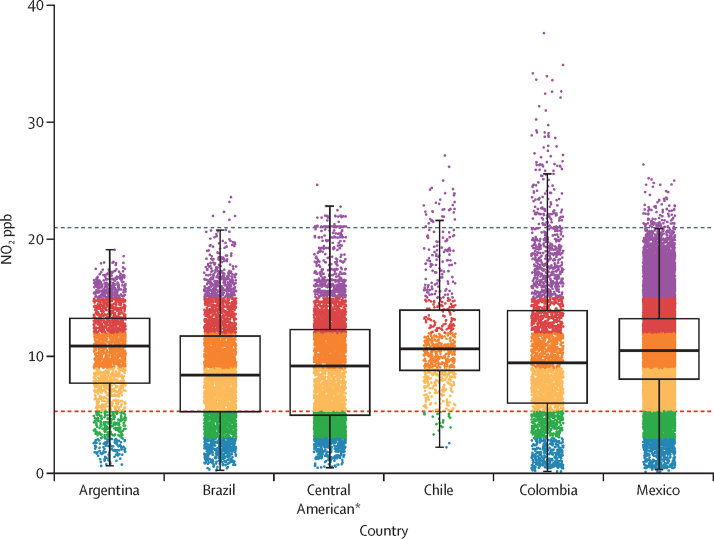
Annual ambient NO_2_ within 47 187 urban neighbourhoods in Latin America Each dot represents annual NO_2_ in one neighbourhood. The red dashed line represents the 2021 WHO guidelines for annual NO_2_ (10 μg/m^3^; approximately 5·3 ppb). The grey dashed line represents the pre-2021 guideline for annual NO_2_ (40 μg/m^3^; approximately 21 ppb), for reference. NO_2_=nitrogen dioxide. ppb=parts per billion. *Central American grouping includes urban neighbourhoods in Costa Rica (n=1 city), Guatemala (n=2), and Panama (n=3).

The median neighbourhood annual NO_2_ ppb across all countries was 10·2 ppb, nearly twice the WHO annual guideline of 5·3 ppb (table 2). Median neighbourhood NO_2_ varied between countries, ranging from 8·4 ppb in Brazil to 10·9 ppb in Argentina. We observed substantial variation in NO_2_ concentrations within countries (table 2 and figure 3). For example, in Colombia, the 5th percentile neighbourhood NO_2_ concentration was 1·4 ppb while the 95th percentile concentration was 21·0 ppb. Across all countries a large proportion of neighbourhoods exceeded WHO guidelines. The highest proportion of neighbourhoods exceeding the guidelines was observed in Chile (723 [97·2%] of 744 neighbourhoods had NO_2_ concentrations above guidelines and 34 [4·6%] of 744 neighbourhoods had NO_2_ concentrations approximately four times greater than guidelines) and lowest in the three study countries in Central America (3939 [70·8%] of 5563 neighbourhoods exceeded guidelines).

**Table 2:** Neighbourhood ambient NO_2_ concentrations and population exposures among 47 187 study neighbourhoods in 326 Latin American cities

	**Study population at census (millions)**	Study population above NO_2_ guidelines*(millions)	Percentage of study population above NO_2_ guidelines*	**Mean neighbourhood NO_2_ (ppb)**	**5th percentile neighbourhood NO_2_ (ppb)**	**Median neighbourhood NO_2_ (ppb)**	**95th percentile neighbourhood NO_2_ (ppb)**
Total	236·0	199·5	84·6%	10·3	3·6	10·2	17·2
Argentina	23·7	21·9	91·9%	10·3	2·9	10·9	16·1
Brazil	108·4	84·6	78·0%	8·7	2·2	8·4	16·2
Central American†	6·6	4·7	70·8%	9·0	2·8	9·2	16·2
Chile	2·8	2·7	97·2%	11·6	5·9	10·6	20·8
Colombia	20·4	17·4	85·5%	10·3	1·4	9·4	21·0
Mexico	74·0	68·2	92·2%	10·7	4·9	10·5	17·1

NO_2_=nitrogen dioxide. ppb=parts per billion.

* WHO annual NO_2_ guidelines (10 μg/m^3^; approximately 5·3 ppb).

† Central American grouping includes urban neighbourhoods in Costa Rica (n=1 city), Guatemala (n=2), and Panama (n=3).

Our study area included approximately 236 million residents as of the most recent census, ranging from 2·8 million residents in Chile to 108·4 million residents in Brazil (table 2). Of these 236 million residents, 84·6% of total residents (nearly 200 million people in census year) lived with annual ambient NO_2_ concentrations above WHO guidelines. The percentage of residents living with ambient NO_2_ concentrations above guidelines varied from 97·2% of residents (2·68 million of 2·76 million) in Chilean cities to 70·8% of residents (4·71 million of 6·65 million) in Central American cities. All other countries had large majorities of residents living with ambient NO_2_ that exceeded guidelines (Mexico 92·2% of residents [68·2 million of 74·0 million], Argentina 91·9% [21·9 million of 23·7 million], Colombia 85·5% [17·4 million of 20·4 million], and Brazil 78·0% [84·6 million of 108·4 million]).

Table 3 shows associations of neighbourhood and city-level characteristics with neighbourhood NO_2_ ambient concentrations. Spearman correlation coefficients for all variables are presented in the appendix (p 13) and no covariates had correlation coefficients greater than 0·52. In the multilevel model adjusting for both neighbourhood and city characteristics simultaneously, higher neighbourhood population density (0·06 higher NO_2_ ppb per unit higher population density Z score [95% CI 0·04 to 0·08]) and higher educational attainment (0·64 ppb per unit higher education Z score [0·61 to 0·67]) were associated with higher neighbourhood NO_2_. Conversely, lower neighbourhood NO_2_ was associated with more greenness (–2·22 ppb per unit NDVI Z score [95% CI –2·25 to –2·19]) and greater distance from the city centre (–0·87 ppb per unit distance Z score [–0·90 to –0·85]). In this same model, higher city-level population density, population size, and traffic congestion were associated with higher NO_2_. Our comparison of empty and multivariable models (both adjusted for country group) indicated that 69% of the between-city variability and 79% of the between-neighbourhood variability was explained by the full host of predictors.

**Table 3:** Mean differences in neighbourhood ambient NO_2_ concentration (ppb) associated with a one-unit Z-score increase in neighbourhood-level and city-level features in 47 187 urban neighbourhoods in Latin America

	**Univariable**		**Multivariable (all)**	
	Estimate	95% CI	Estimate	95% CI
**Neighbourhood-level**				
Population density	0·61*	0·58 to 0·65	0·06*	0·04 to 0·08
Education	1·80*	1·77 to 1·84	0·64*	0·61 to 0·67
Intersection density	0·56*	0·53 to 0·59	0·02	−0·01 to 0·04
Greenness	−2·84*	−2·87 to −2·82	−2·22*	−2·25 to −2·19
Distance from city centre	−1·79*	−1·82 to −1·76	−0·87*	−0·90 to −0·85
**City-level**				
Population density	0·47*	0·10 to 0·84	0·34*	0·06 to 0·63
Education	1·55*	1·12 to 1·98	−0·05	−0·33 to 0·42
Intersection density	0·63*	0·33 to 0·93	0·08	−0·15 to 0·30
Greenness or vegetation	−0·88*	−1·16 to −0·61	0·08	−0·14 to 0·30
Population size	0·76*	0·53 to 1·00	0·31*	0·11 to 0·51
GDP	0·21	−0·05 to 0·47	−0·04	−0·23 to 0·16
Congestion	1·33*	0·99 to 1·68	0·55*	0·23 to 0·87

All independent variables have been Z-transformed using the distribution of all study neighbourhoods or cities, respectively. Univariable and multivariable models were adjusted for country group and have a random intercept for city. Multivariable results are from multilevel models that incorporate neighbourhood and city variables simultaneously. GDP=gross domestic product. NO_2_=nitrogen dioxide. ppb=parts per billion.

* These estimates represent coefficients with statistical significance of p<0·05.

In the sensitivity analysis using the same multivariable model but replacing NO_2_ and urban features in or closest to 2019 with NO_2_ and urban features in or closest to each country's census year, we found similar associations in direction and magnitude in the main and sensitivity analyses (appendix p 12). When stratifying by country, there were consistent associations across all countries between higher NO_2_ and higher neighbourhood educational attainment and between lower NO_2_ and neighbourhoods that were greener and more distant from the city centre (appendix p 11). In contrast, the associations between NO_2_ and population and intersection density varied across countries (appendix p 11).

## Discussion

We performed a highly spatially resolute descriptive analysis among 236 million residents of over 47 000 urban neighbourhoods in 326 Latin America cities, examining (1) population exposure to ambient NO_2_ and (2) associations between neighbourhood ambient NO_2_ concentrations and population characteristics and urban form. We found four key findings. First, nearly nine out of ten residents, or around 200 million people, are exposed to ambient NO_2_ concentrations that exceed the current WHO guidelines. Second, we found that NO_2_ variability was widest within cities rather than between cities or countries. Third, larger, denser, and more congested cities had higher NO_2_. Last, within cities, we found that neighbourhoods with less vegetation and closer to the city centre had higher NO_2_. These findings highlight the magnitude of harmful human exposure to ambient NO_2_ in cities across Latin America, reveal important within-city differences in NO_2_ exposures, and highlight potential interventions that might reduce exposures to this harmful urban air pollutant.

We found a mean NO_2_ concentration of 10·3 ppb at the neighbourhood level in 2019. We are not aware of other regional analyses of neighbourhood-level NO_2_ or within-city variation of NO_2_ in Latin America, yet the concentrations we observed are similar to a city-level analysis by Anenberg and colleagues using the same NO_2_ source, that found an overall population-weighted NO_2_ concentration of 10·6 ppb across urban Latin America in the same year.^11^ Anenberg and colleagues estimated that urban NO_2_ across Latin America was higher than urban areas of sub-Saharan Africa (7·1 ppb) and similar to urban south Asia (10·1 ppb) and high-income countries (11·1 ppb). Given the 75% reduction in annual NO_2_ guidelines in the 2021 WHO air quality guidelines,^8^ urban populations worldwide find themselves with long-term NO_2_ concentrations above the updated guidelines established to protect public health. Our highly spatially granular analysis of urban Latin America finds that Latin American cities are no exception, with 85% (199·5 million) of the 236 million residents in our study area living in neighbourhoods with NO_2_ concentrations above WHO guidelines.

Few studies have examined the substantial within-city variation in population exposure to ambient NO_2_ and the associations of urban and population characteristics with neighbourhood differences in NO_2_ concentrations within Latin America. Understanding the drivers of between-city and within-city differences in ambient NO_2_ exposure is critical to design policies that promote health and health equity in this highly urbanised region. Our study is unique in its breadth (all cities of 100 000 residents or more in eight countries) and in our examination of how neighbourhood and city level factors independently relate to urban NO_2_ exposure. We found that higher population density at both the city and neighbourhood levels were independently associated with higher NO_2_, although the effect size was much greater at the city level than the neighbourhood level. This is consistent with regional studies in the USA^4^ and Europe^20^ and emphasises the nature of NO_2_ pollution as a spatially varying byproduct of local anthropogenic fossil fuel combustion. In models adjusted for city and neighbourhood-level factors, we found that greenness at the neighbourhood-level, but not city-level, was associated with lower neighbourhood NO_2_. Overall, cities that had more vehicular traffic congestion tended to have higher NO_2_ concentrations. This is unsurprising given the continued dominance of fossil fuels for motor vehicles and transit, and the role of fossil fuel motorised transit in generating NO_2_.^1^ Taken together, these findings suggest that reducing city-level congestion (of fossil-fuel powered vehicles) and increasing neighbourhood greenness warrant further attention as potential actionable environmental interventions to reduce population exposure to NO_2_ in urban areas.

Our findings emphasise the need for context-specific analyses of pollution exposures among populations in the Global South, where population patterns in urban areas and environmental justice concerns might be distinct from better-studied cities in high-income countries.^21^ Specifically, we found an unexpected positive association between neighbourhood educational attainment and neighbourhood ambient NO_2_ (ie, neighbourhoods with higher education experience higher NO_2_ concentrations), which was consistent within each country in a stratified sensitivity analysis. This corroborates a study of São Paulo,^22^ but contrasts with multicity studies of neighbourhood ambient NO_2_ in Europe^23^ and the USA.^4^ In these high-income settings, ambient NO_2_ is often higher in lower socioeconomic status (SES) areas, driven largely by neighbourhood urbanicity and proximity to highways. In Europe, this association has been observed to be heterogenous across cities, suggesting the importance of within-city analyses that capture local disparities.^23^ Contrasting associations between neighbourhood SES and ambient air pollutants might reflect different patterns of residential segregation, specifically how segregation by SES is distributed between the urban core and urban periphery. However, in many Latin American cities, regardless of air pollution in their residential neighbourhoods, individuals with lower SES experience substantially higher personal exposure to air pollution due to longer times spent commuting on roads with high levels of traffic-related air pollutants,^24^ such as NO_2_. Furthermore, individuals living in lower SES areas might experience greater health impacts of air pollution compared to those in higher SES areas due to a higher prevalence of chronic conditions and lower access to medical care.^21^, ^25^ Quantifying population exposures at the place of residence is only one step in the process of understanding the true impact of NO_2_ exposure on health disparities.^21^

This study has some limitations. While we examined congestion at the city level and intersection density at the city and neighbourhood levels, we were unable to look at traffic volume within the city or differences in vehicle fleet age or fuel type, which is a major driver of intra-urban variation in NO_2_ at the neighbourhood level.^26^ Fossil-fuel powered vehicles are a major source of ambient NO_2_ and limiting their use through policies that support electric vehicles and public transit warrants critical attention as potential interventions to reduce urban NO_2_. Studies of additional urban design and transit features are needed to further clarify potential interventions. We were also unable to include measures of indoor exposures to NO_2_ generated by indoor or household burning of fossil fuels, such as gas appliances, which is a major source of NO_2_ exposure.^27^, ^28^ In Latin America, indoor air pollution exposure from household cooking and heating varies by urbanicity and SES^28^, ^29^ and it is likely that there are intra-urban and inter-urban differences in personal exposure to NO_2_ that are not captured by our measures of residential ambient NO_2_. Furthermore, while we examined neighbourhood ambient NO_2_ and urban form from 2019, we used neighbourhood-level population data from the most recent census for each country, with a mode census year of 2010 (appendix p 2). Given larger regional trends in population growth and urbanisation,^9^ we expect that our estimates of the number of population exposed are underestimates given the urban population has probably grown since the last census year. However, our sensitivity analysis of associations between NO_2_ and urban features showed similar findings between the main model examining NO_2_ and urban features in (or closest to) 2019 and the sensitivity analysis model using NO_2_ and urban features in (or closest to) the census year (appendix p 12). Furthermore, while we consider incorporating subnational estimates of GDP to be a strength of the study, these estimates were available at the departmental or state level only. This introduced measurement error and could have impacted our findings for GDP. Finally, NO_2_ estimates were derived from models that incorporate satellite imagery and air monitors, yet Latin America is heavily under-represented in the underlying models due to the scarcity of NO_2_ monitors within the region. While South America outperformed all other regions in validation analyses of the underlying land use models, strengthening NO_2_ air monitor networks in the region is needed to improve regional estimates and address the wide disparities in air pollution measurement between the Global South and Global North.

Our study reports on an unprecedented analysis of over 47 000 neighbourhoods across this highly urbanised region of the Global South. By compiling small area census records with a new, spatially resolved estimate of ambient NO_2_, this analysis provides novel and practical evidence of within-city differences in neighbourhood-level population exposure to NO_2_ in the context of recently updated WHO guidelines. This evidence can support urban policy makers and practitioners by guiding the development of policies and interventions that reduce urban NO_2_ exposure by targeting specific features of the urban environment in Latin America and beyond.

In conclusion, among 236 million residents of over 47 000 neighbourhoods in 326 Latin American cities, nearly nine in ten people live in neighbourhoods with ambient NO_2_ concentrations that exceed WHO guidelines. Neighbourhoods that are denser, closer to the urban core, and have less vegetation have higher concentrations of NO_2_, compared with less dense neighbourhoods in the urban periphery. Cities with higher vehicle congestion, population size, and population density have higher concentrations of ambient NO_2_. Our findings suggest that (1) increasing neighbourhood-level greenness and (2) reducing city-level pollution from fossil fuel-powered vehicles by promoting active and public transit and vehicle fleet electrification have potential as actionable interventions to reduce ambient NO_2_ exposures in Latin American cities.

## Data sharing

Vital registration and population data for Brazil, Chile, and Mexico were downloaded from publicly available repositories of statistical agencies in each country. Vital registration and population data for Argentina, Costa Rica, Guatemala, and Panama were obtained directly from statistical agencies in each country. A link to these agency websites can be accessed via https://drexel.edu/lac/data-evidence/data-acknowledgements. Global NO_2_ concentrations are available at: https://doi.org/10.6084/m9.figshare.12968114 as described here: https://doi.org/10.1016/S2542-5196(21)00255-2. Please contact the corresponding author about access to neighbourhood-level urban features.

## Declaration of interests

We declare no competing interests.
