# Supplementary material for: Ambient nitrogen dioxide in 47 187 neighbourhoods across 326 cities in eight Latin American countries: population exposures and associations with urban features
Source: Lancet Planet Health. 2023 Dec 4;7(12):e976–84. doi: 10.1016/S2542-5196(23)00237-1 (PMC10716820; doi:10.1016/S2542-5196(23)00237-1)
Supplement: Supplementary appendix [file mmc1.pdf]

THE LANCET
Planetary Health

Supplementary appendix

This appendix formed part of the original submission and has been peer reviewed. We post it as supplied by the authors.

Supplement to: Kephart JL, Gouveia N, Rodríguez DA, et al. Ambient nitrogen dioxide in 47 187 neighbourhoods across 326 cities in eight Latin American countries: population exposures and associations with urban features. *Lancet Planet Health* 2023; **7:** e976–84.

Ambient nitrogen dioxide in 47,187 neighborhoods across 326 cities in eight Latin American countries: population exposures and associations with urban features

Supplementary Material

Supplemental Table S1: Neighborhood administrative units and years of most recent available census.

Supplemental Table S2: Observed neighborhood count and population for 326 study cities.

Supplemental Table S3: Sensitivity analysis of multilevel associations between neighborhood NO_2 and urban features, stratified by country.

Supplemental Figure S1: Comparison of estimates from main analysis (using available data closest to 2019) with sensitivity analysis (using available data closest to census year).

Supplemental Figure S2: Spearman correlation coefficients between variables in multilevel analysis of associations between neighborhood NO_2 and features of the urban environment.

Appendix I: Detailed descriptions of urban environmental measures and their interpretation

Supplemental Table S1: Neighborhood administrative units and years of most recent available census.

Country	Administrative unit (neighborhood)	Census/population year
Argentina	Fraccion Censal	2010
Brazil	Áreas de Ponderação	2010
Chile	Zona Censal	2017
Colombia	Sector Urbano, Clase = 1	2018
Costa Rica	Distrito	2011
Guatemala	Sector Censal	2002
Mexico	Área Geoestadística Básica	2010
Panama	Barrio	2010

Supplemental Table S2: Observed neighborhood count and population for 326 study cities.

Country	City name	Number of neighborhoods observed	Observed city population (census year)	Median neighborhood population
Argentina	Bahia Blanca	32	301,572	8,720
	Buenos Aires	1,261	14,589,040	10,240
	Comodoro Rivadavia	19	186,583	10,627
	Concordia	16	170,033	9,066
	Cordoba	120	1,554,755	13,055
	Corrientes	23	358,223	16,518
	Formosa	22	234,354	7,809
	Jujuy	21	317,880	16,969
	Mar del Plata	83	618,989	6,917
	Rawson-Trelew	13	131,313	12,550
	Rio Cuarto	29	246,393	8,487
	Rosario	105	1,350,860	13,039
	Salta	33	579,665	18,095
	San Carlos de Bariloche	19	133,500	7,191
	San Juan	41	503,946	12,155
	San Luis	19	204,019	13,182
	San Miguel de Tucuman-Tafi Viejo	66	994,553	13,591
	San Nicolas de los Arroyos	21	145,857	8,762
	Santa Fe	35	525,093	13,284
	Santiago del Estero	34	409,404	14,191
	Tandil	22	123,871	4,377
	Villa Mercedes	18	125,899	5,417
Brazil	Alagoinhas	7	141,949	17,151
	Anapolis	13	337,888	25,467
	Angra dos Reis	7	169,511	19,945
	Apucarana	7	120,919	14,107
	Aracaju	29	835,816	28,110
	Aracatuba	11	181,579	13,996
	Araguaina	7	150,484	22,438
	Araguari	6	109,801	18,500
	Arapiraca	12	214,006	15,554
	Arapongas	9	162,012	19,986
	Araraquara	17	243,140	14,588
	Araras	7	118,843	16,974
	Araruama	11	209,093	18,914
	Atibaia	8	146,311	14,386
	Balneario Camboriu	10	170,450	15,271
	Barbacena	7	126,284	14,254
	Barreiras	7	137,427	18,226

Country	City name	Number of neighborhoods observed	Observed city population (census year)	Median neighborhood population
Brazil	Barretos	6	112,101	17,976
	Bauru	17	343,937	20,070
	Belem	65	2,101,883	32,621
	Belo Horizonte	174	4,707,362	26,724
	Bento Goncalves	11	163,159	13,814
	Birigui	6	108,728	16,865
	Blumenau	29	486,379	15,648
	Boa Vista	14	284,313	19,798
	Botucatu	7	127,328	17,199
	Braganca Paulista	9	146,744	14,818
	Brasilia	78	3,187,984	40,502
	Brusque	6	123,933	19,340
	Cabo Frio	14	301,817	19,033
	Cachoeiro de Itapemirim	9	189,889	17,057
	Campina Grande	25	465,064	16,940
	Campinas	115	2,814,738	22,178
	Campo Grande	8	786,797	103,857
	Campos dos Goytacazes	9	463,731	26,514
	Caraguatatuba	10	174,782	14,628
	Caruaru	12	314,912	15,682
	Cascavel	17	286,205	15,861
	Castanhal	8	173,149	17,211
	Catanduva	7	112,820	14,667
	Caxias	7	155,129	17,917
	Caxias do Sul	27	435,564	14,073
	Chapeco	11	183,530	16,573
	Conselheiro Lafaiete	5	116,512	22,975
	Criciuma	19	331,281	16,357
	Cuiaba	35	803,694	20,735
	Curitiba	100	2,857,466	28,032
	Divinopolis	7	213,016	34,904
	Dourados	10	196,035	18,931
	Feira de Santana	16	589,925	36,154
	Florianopolis	57	851,955	13,678
	Fortaleza	99	3,411,846	32,645
	Foz do Iguacu	11	256,088	14,910
	Franca	21	318,640	14,961
	Garanhuns	7	129,408	16,084
	Goiania	69	2,013,288	29,664
	Governador Valadares	13	263,689	19,860
	Guarapari	6	105,286	16,407

Country	City name	Number of neighborhoods observed	Observed city population (census year)	Median neighborhood population
Brazil	Guarapuava	9	167,328	18,607
	Guaratingueta	11	249,013	19,657
	Ilheus	9	184,236	18,385
	Imperatriz	15	290,946	18,209
	Ipatinga	26	536,909	19,363
	Itabira	5	109,783	23,020
	Itabuna	14	204,667	14,584
	Itajai	16	308,534	16,147
	Itapetininga	7	144,377	20,768
	Jaragua do Sul	11	193,611	16,029
	Jau	7	131,040	16,552
	Jequie	7	151,895	21,372
	Ji-Parana	6	116,610	16,053
	Joao Pessoa	27	1,034,615	26,730
	Joinville	30	540,098	16,847
	Juazeiro do Norte	21	426,690	17,859
	Juiz de Fora	14	516,247	35,200
	Jundiai	34	596,148	15,216
	Lages	9	156,727	17,793
	Limeira	7	296,051	43,443
	Linhares	7	141,306	19,684
	Londrina	13	651,632	28,202
	Macae	12	206,728	16,648
	Macapa	22	499,466	20,183
	Maceio	28	1,061,809	32,531
	Manaus	33	1,802,014	50,359
	Maraba	4	233,669	57,743
	Marilia	10	216,745	21,093
	Maringa	29	507,819	17,076
	Mogi Guacu	13	233,794	16,622
	Montes Claros	22	361,915	15,740
	Mossoro	13	259,815	19,107
	Natal	43	1,227,675	28,899
	Nova Friburgo	9	182,082	15,964
	Ourinhos	5	103,035	20,664
	Palmas	10	228,332	19,111
	Paranagua	7	140,469	15,886
	Parauapebas	7	153,908	21,647
	Parnaiba	5	145,705	27,949
	Parobe	8	161,653	19,392
	Passo Fundo	10	184,826	16,249

Country	City name	Number of neighborhoods observed	Observed city population (census year)	Median neighborhood population
Brazil	Passos	6	106,290	15,060
	Patos de Minas	9	138,710	14,087
	Pelotas	21	352,573	16,052
	Petrolina	25	491,927	18,274
	Petropolis	15	295,917	19,105
	Piracicaba	20	364,571	18,116
	Pocos de Caldas	9	152,435	13,738
	Ponta Grossa	15	311,611	21,495
	Porto Alegre	163	3,581,951	19,206
	Porto Seguro	7	126,929	16,062
	Porto Velho	23	448,306	18,202
	Pouso Alegre	6	130,615	18,675
	Presidente Prudente	13	231,123	15,309
	Recife	116	3,513,174	29,687
	Resende	8	165,144	18,708
	Ribeirao Preto	19	604,682	31,546
	Rio Branco	8	336,038	42,495
	Rio Claro	11	207,887	17,868
	Rio Grande	11	197,228	15,784
	Rio Verde	8	176,424	18,367
	Rio das Ostras	9	141,023	13,287
	Rio de Janeiro	332	11,737,101	27,907
	Rondonopolis	11	195,476	16,522
	Salvador	88	3,200,122	29,702
	Santa Cruz do Sul	6	118,374	17,359
	Santa Maria	16	261,031	14,007
	Santarem	11	294,580	28,791
	Santos	59	1,616,491	24,946
	Sao Carlos	14	221,950	14,376
	Sao Jose do Rio Preto	27	484,625	16,982
	Sao Jose dos Campos	25	925,887	30,461
	Sao Luis	41	1,309,330	32,946
	Sao Paulo	621	19,411,753	30,332
	Sertaozinho	6	110,074	16,290
	Sete Lagoas	13	214,152	15,963
	Sobral	9	188,233	18,143
	Sorocaba	26	722,733	27,540
	Tatui	4	107,326	29,668
	Taubate	23	466,665	16,656
	Teixeira de Freitas	6	138,341	20,102
	Teofilo Otoni	6	134,745	21,714

Country	City name	Number of neighborhoods observed	Observed city population (census year)	Median neighborhood population
Brazil	Teresina	23	969,690	31,055
	Teresopolis	9	163,746	15,025
	Toledo	7	119,313	13,813
	Tubarao	8	129,544	13,938
	Uberaba	16	295,988	18,179
	Uberlandia	17	604,013	32,159
	Uruguaiana	6	125,435	16,676
	Varginha	7	123,081	17,021
	Vitoria	72	1,565,393	19,349
	Vitoria da Conquista	14	306,866	21,101
	Vitoria de Santo Antao	6	129,974	23,816
	Volta Redonda	26	553,113	20,032
Chile	Antofagasta	39	175,736	4,201
	Arica	48	148,845	2,799
	Calama	17	82,407	4,853
	Chillan	17	64,808	3,884
	Concepcion	70	244,589	3,396
	Copiapo	28	103,977	3,612
	Curico	15	51,364	2,977
	Iquique	43	183,465	4,351
	La Serena-Coquimbo	33	145,735	4,475
	Los Angeles	11	46,679	3,827
	Osorno	12	42,874	3,339
	Puerto Montt	19	54,339	2,995
	Punta Arenas	37	114,473	3,035
	Quillota	9	32,508	3,433
	Rancagua	22	74,257	2,957
	San Antonio	6	23,025	3,731
	Santiago	168	679,962	4,061
	Talca	23	76,911	3,349
	Temuco	24	83,093	2,867
	Valdivia	23	86,121	3,719
	Valparaiso-Vina del Mar	80	244,530	3,215
Colombia	Armenia	71	269,254	2,242
	Barrancabermeja	68	147,544	1,700
	Barranquilla	197	1,615,325	6,489
	Bogota	518	5,542,505	8,392
	Bucaramanga	122	777,270	5,120
	Buenaventura	57	324,207	3,283
	Buga	21	86,086	3,739
	Cali	363	2,174,657	3,929

Country	City name	Number of neighborhoods observed	Observed city population (census year)	Median neighborhood population
Colombia	Cartagena	116	711,222	4,944
	Cartago	20	101,209	2,743
	Cucuta	142	615,901	3,218
	Duitama	15	90,794	5,875
	Florencia	34	137,896	3,270
	Fusagasuga	15	107,259	5,713
	Girardot	36	93,478	1,844
	Ibague	70	420,249	4,138
	Manizales	98	372,114	2,839
	Medellin	362	3,074,734	6,738
	Monteria	58	344,927	4,018
	Neiva	88	319,335	2,460
	Palmira	48	249,493	4,340
	Pasto	39	383,846	9,523
	Pereira	47	525,248	11,430
	Popayan	32	258,653	7,891
	Quibdo	8	15,332	714
	Riohacha	60	152,530	1,670
	Santa Marta	102	433,784	2,374
	Sogamoso	17	120,320	6,553
	Tulua	58	161,534	1,554
	Tunja	29	152,868	4,835
	Valledupar	86	308,771	1,303
	Villavicencio	41	276,567	4,733
	Yopal	28	75,543	913
Costa Rica	San Jose	169	2,207,560	9,727
Guatemala	Guatemala City	3,602	2,458,830	663
	Quetzaltenango	250	201,801	803
Mexico	Acapulco de Juarez	519	863,346	1,279
	Acayucan	59	112,996	998
	Acuna	108	136,755	1,176
	Aguascalientes	327	932,369	2,285
	Campeche	124	259,005	1,812
	Cancun	361	676,832	1,498
	Celaya	435	775,761	439
	Chetumal	147	244,553	1,078
	Chihuahua	639	852,527	1,227
	Chilpancingo	191	241,717	560
	Ciudad Valles	111	167,680	1,102
	Ciudad del Carmen	74	221,094	2,584
	Coatzacoalcos	143	346,177	2,337

Country	City name	Number of neighborhoods observed	Observed city population (census year)	Median neighborhood population
Mexico	Colima	237	334,240	1,241
	Cordoba	113	316,032	2,070
	Cuauhtemoc	134	154,639	904
	Cuautla	236	434,147	1,207
	Cuernavaca	456	924,964	1,709
	Culiacan	441	858,638	1,677
	Delicias	103	137,935	913
	Durango	384	582,264	1,092
	Ensenada	336	466,814	981
	Fresnillo	186	213,139	398
	Guadalajara	1,677	4,434,866	2,456
	Guanajuato	115	171,706	629
	Guaymas	169	203,419	954
	Hermosillo	518	784,339	1,121
	Hidalgo del Parral	76	107,061	1,460
	Iguala	159	140,363	367
	Irapuato	166	529,440	2,172
	Juarez	606	1,328,636	1,929
	La Paz	228	251,871	721
	La Piedad	96	249,512	1,256
	Leon	598	1,609,393	1,932
	Los Mochis	245	416,299	1,235
	Manzanillo	118	155,537	954
	Matamoros	290	489,190	1,414
	Mazatlan	233	438,434	1,749
	Merida	557	973,031	1,590
	Mexicali	463	936,826	1,637
	Mexico City	5,687	20,116,842	3,179
	Minatitlan	140	356,137	2,063
	Monclova	205	317,313	1,499
	Monterrey	1,644	4,161,267	2,414
	Morelia	391	829,622	1,598
	Navojoa	98	157,729	1,183
	Nogales	167	220,292	940
	Nuevo Laredo	226	384,030	1,390
	Oaxaca de Juarez	375	607,963	1,553
	Obregon	254	409,310	1,083
	Ocotlan	80	141,375	1,068
	Orizaba	192	427,406	1,871
	Pachuca de Soto	360	512,196	1,077
	Piedras Negras	156	180,734	1,036

Country	City name	Number of neighborhoods observed	Observed city population (census year)	Median neighborhood population
Mexico	Playa del Carmen	95	159,310	998
	Poza Rica de Hidalgo	215	513,518	1,657
	Puebla de Zaragoza	1,078	2,855,849	2,395
	Puerto Vallarta	323	379,886	769
	Queretaro	400	1,097,025	2,097
	Reynosa	392	727,147	1,489
	Rio Verde	48	135,452	1,451
	Salamanca	92	260,732	1,930
	Saltillo	393	823,128	1,873
	San Cristobal de las Casas	63	185,914	2,616
	San Francisco del Rincon	74	182,365	710
	San Juan Bautista Tuxtepec	57	155,766	1,798
	San Juan del Rio	93	241,699	1,907
	San Luis Potosi	389	1,040,443	2,590
	San Luis Rio Colorado	232	178,380	786
	Santo Domingo Tehuantepec	117	161,337	1,185
	Tampico	405	859,416	1,910
	Tapachula	164	320,451	1,267
	Tecoman	100	141,421	1,277
	Tehuacan	127	296,899	2,149
	Tepic	206	429,351	1,809
	Teziutlan	41	122,500	2,339
	Tianguistenco	57	157,944	1,811
	Tijuana	701	1,751,430	2,261
	Tlaxcala	302	499,567	1,365
	Toluca	566	2,014,091	2,752
	Torreon	672	1,215,814	1,507
	Tula de Allende	161	286,424	1,250
	Tulancingo de Bravo	125	239,579	1,344
	Tuxtla Gutierrez	266	684,156	2,331
	Uriangato	119	108,669	96
	Uruapan	163	315,350	1,616
	Veracruz	368	811,671	1,977
	Victoria	176	321,953	1,603
	Villahermosa	177	755,425	3,012
	Xalapa	246	666,532	2,226
	Zacatecas	303	309,660	735
	Zamora	119	250,113	1,862
Panama	Colon	160	200,278	543
	David	304	196,983	290
	Panama City	1,087	1,391,754	549

Supplemental Table S3: Sensitivity analysis of multilevel associations between neighborhood NO_2 and urban features, stratified by country. Mean differences in neighborhood ambient NO2 concentration (ppb) associated with a one-unit z-score increase in neighborhood- and city-level features in five countries in Latin America. Guatemala, Costa Rica, and Panama are not included due to the small number of cities (≤3) in each of these countries.

		All Countries (main analysis)	Brazil	Argentina	Chile	Colombia	Mexico
Neighborhood-Level	Population density*	**0.06 (0.04 to 0.08)**	**0.78 (0.56 to 0.99)**	**0.54 (0.39 to 0.68)**	**0.38 (0.05 to 0.70)**	**-0.50 (-0.68 to -0.31)**	-0.06 (-0.14 to 0.01)
	Education*	**0.64 (0.61 to 0.67)**	**0.86 (0.77 to 0.96)**	**1.12 (0.96 to 1.27)**	**1.07 (0.70 to 1.47)**	**1.06 (0.92 to 1.19)**	**0.58 (0.53 to 0.63)**
	Intersection density*	0.02 (-0.01 to 0.04)	**0.72 (0.29 to 1.16)**	**2.39 (1.93 to 2.86)**	**-0.62 (-0.99 to -0.24)**	**0.06 (0.03 to 0.09)**	**-0.19 (-0.25 to -0.13)**
	Greenness*	**-2.22 (-2.25 to -2.19)**	**-2.56 (-2.68 to -2.44)**	**-1.81 (-1.94 to -1.68)**	**-1.87 (-2.12 to -1.60)**	**-2.13 (-2.32 to -1.94)**	**-2.33 (-2.38 to -2.29)**
	Distance from city center*	**-0.87 (-0.90 to -0.85)**	**-0.71 (-0.78 to -0.64)**	**-0.81 (-0.88 to -0.74)**	**-2.60 (-2.97 to -2.24)**	**-0.56 (-0.76 to -0.37)**	**-0.84 (-0.87 to -0.81)**
City-level	Population density*	**0.34 (0.06 to 0.63)**	-0.13 (-0.55 to 0.29)	-0.87 (-3.32 to 1.58)	-0.93 (-2.15 to 0.31)	**1.63 (0.91 to 2.35)**	-0.09 (-0.56 to 0.37)
	Education*	0.05 (-0.33 to 0.42)	0.00 (-0.5 to 0.51)	0.19 (-1.91 to 2.28)	0.63 (-2.77 to 4.04)	**3.32 (0.19 to 6.45)**	-0.43 (-0.90 to 0.03)
	Intersection density*	0.08 (-0.15 to 0.30)	0.20 (-0.19 to 0.58)	0.22 (-1.14 to 1.57)	0.21 (-0.91 to 1.30)	**-1.14 (-1.95 to -0.34)**	0.13 (-0.14 to 0.41)
	Greenness/vegetation*	0.08 (-0.14 to 0.30)	-0.09 (-0.74 to 0.56)	0.29 (-0.25 to 0.83)	0.27 (-0.12 to 0.66)	3.61 (-0.17 to 7.39)	-0.01 (-0.28 to 0.25)
	Population size*	**0.31 (0.11 to 0.51)**	**0.34 (0.09 to 0.59)**	0.37 (-0.09 to 0.84)	**3.03 (2.20 to 3.85)**	**-1.85 (-3.52 to -0.19)**	0.02 (-0.28 to 0.32)
	GDP*	-0.04 (-0.23 to 0.16)	0.09 (-0.28 to 0.47)	-0.29 (-1.57 to 1.00)	0.12 (-0.41 to 0.67)	0.05 (-2.2 to 2.29)	-0.06 (-0.25 to 0.13)
	Congestion*	**0.55 (0.23 to 0.87)**	0.17 (-0.47 to 0.8)	0.45 (-1.94 to 2.85)	0.30 (-0.69 to 1.30)	1.03 (-0.11 to 2.17)	**1.11 (0.56 to 1.67)**

*All independent variables have been z-transformed using the distribution of all study neighborhoods or cities, respectively. Estimates in bold font represent coefficients with statistical significance of $p<0.05$.

Supplemental Figure S1: Difference in neighborhood NO_2 ppbv per unit higher z-score for each covariate, comparing main analysis (using available data closest to 2019) with sensitivity analysis (using available data closest to census year). Vertical and horizontal lines indicate 95% confidence intervals.

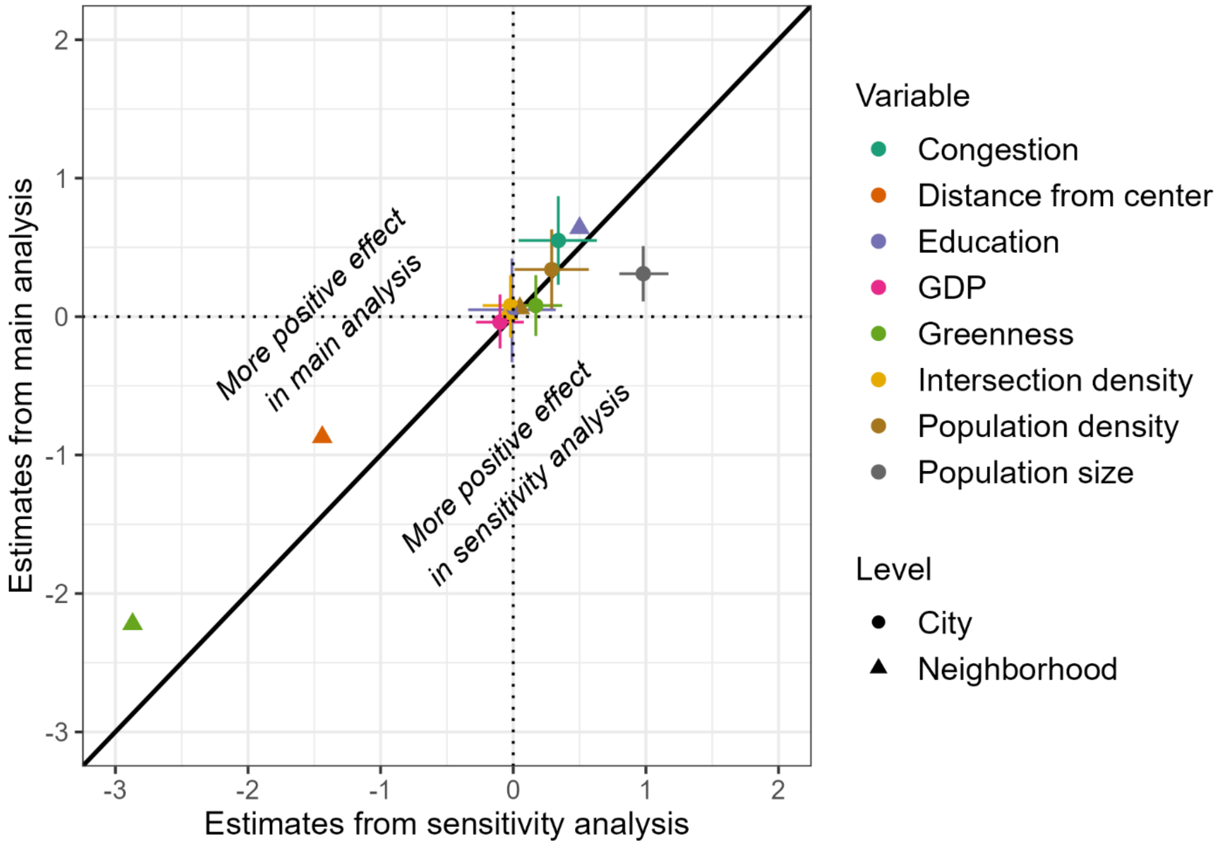

Supplemental Figure S2: Spearman correlation coefficients between variables in multilevel analysis of associations between neighborhood NO_2 and features of the urban environment.

	NO2	Neighb. pop. density	Neighb. educ.	Neighb. int. density	Neighb. green	Neighb. distance	City pop. density	City educ.	City int. density	City green	City pop. size	City GDP	City congestion
NO2		Corr: 0.229	Corr: 0.327	Corr: 0.202	Corr: -0.662	Corr: -0.292	Corr: 0.171	Corr: 0.197	Corr: 0.185	Corr: -0.226	Corr: 0.293	Corr: 0.082	Corr: 0.247
Neighb. pop. density			Corr: -0.012	Corr: 0.131	Corr: -0.178	Corr: -0.091	Corr: 0.248	Corr: 0.067	Corr: 0.051	Corr: 0.060	Corr: 0.153	Corr: 0.033	Corr: 0.263
Neighb. educ.				Corr: 0.107	Corr: -0.418	Corr: -0.128	Corr: -0.235	Corr: 0.433	Corr: -0.006	Corr: -0.236	Corr: 0.133	Corr: 0.128	Corr: -0.113
Neighb. int. density					Corr: -0.198	Corr: -0.122	Corr: 0.147	Corr: 0.109	Corr: 0.174	Corr: -0.012	Corr: 0.006	Corr: -0.032	Corr: 0.076
Neighb. green						Corr: 0.185	Corr: 0.107	Corr: -0.299	Corr: -0.183	Corr: 0.517	Corr: -0.198	Corr: -0.138	Corr: -0.022
Neighb. distance							Corr: 0.005	Corr: 0.092	Corr: -0.142	Corr: -0.091	Corr: 0.309	Corr: 0.149	Corr: 0.080
City pop. density								Corr: 0.149	Corr: 0.220	Corr: 0.329	Corr: 0.336	Corr: -0.045	Corr: 0.720
City educ.									Corr: 0.119	Corr: -0.293	Corr: 0.268	Corr: 0.259	Corr: 0.350
City int. density										Corr: -0.132	Corr: -0.192	Corr: -0.095	Corr: 0.094
City green											Corr: -0.031	Corr: -0.077	Corr: 0.210
City pop. size												Corr: 0.268	Corr: 0.507
City GDP													Corr: 0.110
City congestion													

Appendix I: Detailed descriptions of urban environmental measures and their interpretation

Population (neighborhood/city). Number of residents reported by most recent available national census (see Supplemental Table S1 for census year for each country).

Population Density (neighborhood/city). Population (in thousands) per square kilometer of neighborhood/city area. Population sourced from national censuses.

Educational attainment (neighborhood/city). Percent of the population aged 25 years or older who completed primary education or above. Sourced from national censuses.

Intersection Density (neighborhood/city). The number of intersections per area of city/neighborhood built-up area in square kilometers. Intersections were extracted from street network OpenStreetMap data and included any intersections with >2 connected streets (i.e., cul-de-sacs and road bends represented as a node were excluded). Neighborhood and city indices represent the years of 2020 and 2017, respectively.

Greenness (neighborhood/city). Area median greenness measured by the normalized difference vegetation index (NDVI). NDVI was calculated using MODIS satellite-based observations from the MODIS vegetation product, MOD13Q1.006 for 2015 at a 250 m spatial resolution. We computed the maximum NDVI value for 2019 at 250 m resolution to present the 'greenest' condition of each grid cell within the year, then calculated the median across grid cells contained within each neighborhood.

Distance from city center (neighborhood). Euclidean distance in km between the neighborhood centroid and city hall, based on neighborhood boundaries at time of census.

Gross Domestic Product per Capita (city). City-level GDP in 2011 international US Dollars. Created by Genaioli et al in 2013 and converted into gridded estimates by Kummu et al in 2015. GDP for each year 1990-2015 was estimated by these researchers by modeling data from government, survey and industry. Gridded estimates were matched to SALURBAL cities and GDP was extracted directly from matching administrative units or using population-weighted averages in cases where city boundaries crossed multiple administrative areas. GDP estimates from 2015 (most recent year available) were used.

Gennaioli N, La Porta R, Lopez-de-Silanes F, Shleifer A, Human Capital and Regional Development, The Quarterly Journal of Economics, Volume 128, Issue 1, February 2013, Pages 105–164, https://doi.org/10.1093/qje/qjs050.

Kummu M, Taka M, & Guillaume J. Gridded global datasets for Gross Domestic Product and Human Development Index over 1990–2015. Sci Data 5, 180004 (2018). https://doi.org/10.1038/sdata.2018.4.

Traffic congestion index (city). The increase in travel times due to congestion in the street network. Data sources: The street network was obtained from OpenStreetMap and the travel times from the Google Maps Distance Matrix API. The index for each area unit is approximated by measuring delays for 30 random origin-destination pairs in the street network and seven points in time during peak hours of a typical weekday in 2018. Operationalized as the percent longer trip duration due to traffic congestion, as a percentage of trip time without congestion (possible range, 0 to infinity).
